# Genetic Assessment in the Andean Tropical Fruits *Solanum quitoense* Lam. and *S. betaceum* Cav.: Efforts Towards a Molecular Breeding Approach

**DOI:** 10.3390/plants14060874

**Published:** 2025-03-11

**Authors:** Eduardo Morillo, Johanna Buitron, Denisse Yanez, Pierre Mournet, Wilson Vásquez-Castillo, Pablo Viteri

**Affiliations:** 1 Estación Experimental Santa Catalina, Instituto Nacional de Investigaciones Agropecuarias (INIAP), Quito 170518, Ecuador; eduardo.morillo@iniap.gob.ec (E.M.); johanna.buitron@iniap.gob.ec (J.B.); dayanez@udlanet.ec (D.Y.); pablo.viteri@iniap.gob.ec (P.V.); 2 Ingeniería Agroindustrial y Alimentos, Universidad de Las Américas (UDLA), Quito 170125, Ecuador; 3 Centre de Coopération Internationale en Recherche Agronomique pour le Développement (CIRAD-BIOS), UMR 1334 AGAP, 34398 Montpellier, France; pierre.mournet@cirad.fr

**Keywords:** *Solanum quitoense*, *Solanum betaceum*, Andean fruit crops, SSRs, DNA genotyping

## Abstract

*Solanum quitoense* and *S. betaceum* called, respectively, naranjilla and tomate de arbol, are both tropical Andean fruits of growing interest in the region. Microsatellite primers (SSRs) identified by NGS technology in both species were screened for the development of SSR marker technology. In *S. quitoense*, it was found that 41 primers were successfully transferred to six Lasiocarpa closely related species. Using multiplex primer combinations with the M13-Tailing technology in the DNA analyzer LI-COR 4300s, the variability of these primers in seven *S. quitoense* landraces was characterized. This SSR survey confirmed the narrow genetic base of *S. quitoense* cultivars with the polymorphism of 14 SSR markers. Moreover, transferability rates and genetic diversity analysis revealed a closer genetic relationship between the species *S. candidum* and *S. hirtum* among the Lasiocarpa germplasm screened. On the other hand, 110 SSR primers were screened in four cultivars, segregating plants and wild-related accessions of *S. betaceum*. Polymorphisms for only eight SSR primers were found but including the wild relative *S. unilobum*; in *S. betaceum*, no SSR showed polymorphism confirming the high genetic homogeneity of the cultivars. The results of this study are potentially useful for *S.quitoense* and *S. betaceum* genomics, providing an initial set of SSR markers for molecular characterization in *S. quitoense* germplasm and perspectives for *S. betaceum*.

## 1. Introduction

The species *Solanum quitoense* Lam. (*naranjilla*) and *S. betaceum* Cav. (tomate de árbol or tree tomato) are both important commercial fruit crops for small- and medium-sized farmers in Ecuador and other countries of the region. Both plants are of great potential but require the development of new biotechnology tools to accelerate the processes of germplasm characterization and molecular breeding. *S. quitoense* is a native fruit crop of growing interest in Ecuador and the Andean region (Figure S1A). Taxonomically, it belongs to the section Lasiocarpa, which includes between 11 and 13 species within the Solanaceae family [1]. This fruit is known as “naranjilla” in Ecuador and “lulo” in Colombia. *S. quitoense* represents a crop of high economic potential for the Amazon region in Ecuador, where up to 93% of the national production is located [2]. Nevertheless, in recent years, an appreciable reduction in the crop’s productivity has been noted due to its susceptibility to several diseases and pests, such as fusariosis (*Fusarium oxysporum* species complex), nematodes (*Meloidogine* spp.), and fruit worm (*Neoleucinodes elegantalis, Lepidoptera: Crambidae*) [3,4]. The identification of sources of genetic resistance and the development of new varieties are critical points to be considered for the development of the crop.

Given these circumstances, it is important to characterize and explore the diversity of related species that are sources of genes for *S. quitoense* breeding. The origin of the taxon’s genetic diversity is located in Ecuador, Colombia, and Peru; however, it is estimated that there is low genetic variability in its cultivated form [5,6]. To increase the genetic base of *S. quitoense*, researchers have focused on resistance to pests, which are the main problem for crop production [7]. Many efforts have been reported for the development of interspecific hybrids in order to confer resistance to biotic problems, such as nematodes, but evidence has pointed to a loss of quality in the fruits [2]. Breeding efforts have been supported by morphological and physiological observation; however, there are limitations in the development of breeding programs due to the absence of genetic information. The estimation of the level of polymorphism and the development of specific molecular tools for *S. quitoense* are important scientific requests pointed out by previous studies because only heterologous primers have been tested and polymorphism has rarely been reported [6,8].

*S. betaceum* is a native plant of South America (Figure S1B). Studies show that its most probable center of origin is the jungles and forests of the Tucumano-Bolivian reserve in northwestern Argentina and southern Bolivia. It is considered that the center of domestication of this fruit was in northern Peru and southern Ecuador [9]. The main tree-tomato-producing countries are Ecuador and Colombia. In the former, tree tomato cultivation is carried out mainly by small and medium producers in the provinces of Carchi, Imbabura, Pichincha, Cotopaxi, Tungurahua, Chimborazo, Bolívar, Cañar, Azuay, and Loja [9,10]. In these areas, the tree tomato cultivation area increased by 70% from 2015 to 2017, going from 4500 to 7600 ha [9,11].

The need to improve this fruit tree by breeding programs is essential. A drawback is that breeding periods take longer due to the productive cycle of this fruit crop [10]. Therefore, the use of modern techniques is undoubtedly favorable. Molecular techniques allow increasing knowledge of the distribution of the genetic diversity of different crops, and the selection of potential parents for the breeding program with desirable agronomic characteristics is required [12,13]. Molecular markers are one of the main techniques used to study variability and the traits of interest in crops of agricultural importance [14]. Genetic variability studies have been carried out specifically into tree tomatoes using arbitrary markers, for instance, AFLPs or heterologous SSRs transferred from other species of the genus Solanum [6,15,16,17].

In this study, first, we screened SSR primers from a DNA sequence dataset obtained from Illumina shotgun libraries in *S. quitoense* and *S. betaceum*. We then reported an analysis of SSR diversity in *S. quitoense* and wild close relatives that are currently being used for genetic improvement in Ecuador.

## 2. Materials and Methods

### 2.1. DNA Library and NGS Sequencing

Genomic DNA was isolated from dried leaf samples using a procedure reported by Souza et al. [18]. The biological material used for *S. quitoense* was the local variety “Palora” and “Gigante” for *S. betaceum*.

The DNA library was prepared using the NexteraTM DNA Sample Kit (Illumina, San Diego, CA, USA; Ref. GAO9115). DNA fragmentation started with 50 ng of purified genomic DNA, followed by end-polishing and sequencing adaptor ligation to prepare di-tagged DNA fragment libraries. The quality of the libraries was assessed using a 2100 Bioanalyzer (Agilent, Santa Clara, CA, USA), and the concentration was quantified using a KAPA LibraryqPCR kit (KR0390; Kapa Biosystems, Woburn, MA, USA). Sequencing was performed in an MiSeq Sequencer (Illumina) using the 2 × 300 bp read mode.

### 2.2. Microsatellite Search and Primer Screening

To reduce the raw dataset and maximize the length of the sequences, an assembly of the sequences was performed using the ABySS (Assembly By Short Sequences) assembler [19]. The MIcroSAtellite identification tool (MISA) was used in order to search for microsatellite motifs among the assembled contigs [20]. The search used specific criteria for the design of primers; these included motifs 2–6 bp in size, with a minimum repeat number of four repetitions, and with a maximum difference between two SSRs of 100 bp. The primers would, thus, amplify one or more repeats, effectively encompassing them as the same repeat motif if these were 100 bp or less from each other. For the screening of SSR primers, a set of 100 primer pairs were selected for each species. Sequences containing di- and tri-SSR motifs with a minimum of ten repetitions were retained for the primer survey. SSR contigs were verified beforehand using BLAST v2.7.1. to ensure that they did not match any sequence. For *S. quitoense*, we screened 53 primer pairs corresponding to di-SSR motifs and 47 tri-SSR motifs; for *S. betaceum*, we screened 90 di-SSR motifs and 20 tri-motifs (see Supplementary Materials). The PCR amplification of the expected SSR fragments was first verified on agarose gels. The primers that did not produce a single PCR product or poor amplification were excluded from polymorphism screening.

### 2.3. Polymorphism Screening and DNA Genotyping

For *S. quitoense*, 60 DNA samples of seven landraces were used: 10 plants each from the following varieties: *espinosa*, *agria*, *bolona*, *morada*, *baeza*, and *baeza roja*. We also included 60 DNA samples from six Lasiocarpa species that are being used for interspecific experimental crosses in INIAP: *S. candidum*, *S. hirtum*, *S. pectinatum*, *S. pseudolulo*, *S. sessiliflorum*, and *S. stramonifolium* (see Table 1). For *S. betaceum*, 40 DNA samples were used: 10 plants each from Gigante anaranjado, Gigante morado, Puntón anaranjado, and Puntón morado. We also included five segregating plants from a cross between *S. betaceum* and *S.unilobum*, and five accessions from the wild relative *S. unilobum* from the INIAP genebank (Table 1).

For DNA extractions, we used the protocols described by Souza et al. [18] for leaf tissues and Kang [21] for seeds. DNA was quantified by spectrophotometry and normalized to 5 ng/µL to carry out the PCR assays. Genotyping was performed in an LI-COR 4300s DNA analyzer (Lincoln, NE, USA) using the M13 Tailing methodology according to [22]. PCR amplifications were multiplexed in a 7 µL reaction volume with 10 ng DNA per reaction, 0.01 µM forward primer with M13 tail, 0.16 µM of reverse primer, 0.16 µM of M13 primer with IRDye700 or IRDye 800 fluorescence, 2.5 mM of MgCl_2_, 0.2 mM of dNTP, and 0.25 U of Gotaq DNA polymerase. SSR visualization was carried out using the IRDye fluorescence scanning system and allele size assignment was calculated in SAGA-GT™ v3.3.0 software (LI-COR, Lincoln, NE, USA) with a 50–350 bp molecular size marker IRDye (reference Cat. No. LI-COR 829-05343/44). SSR matrix data were exported to Excel for statistical analysis.

### 2.4. Statistical Analysis

Statistical parameters, such as the number of alleles per locus, observed (Ho) and expected heterozygosities (He), polymorphism information content (PIC) of each locus, and the presence of linkage disequilibrium (LD) were calculated with POWER MARKER 3.25 [23]. Multivariate analyses (PCO) were performed based on the Jaccard similarity coefficient and NJ method using the NTSYS-pc v2.02 program [24]. Unrooted majority rule consensus tree and bootstrap analysis were performed using 1000 replicates with the Phylip package’s Consensus tree program, version 3.6a3 [25].

## 3. Results

### 3.1. NGS Sequencing Analysis and In Silico SSR Identification

For *S. quitoense*, a total number of 1,400,090 sequences were examined, of which 31,759 assembled contigs were identified as containing SSR motifs. For *S. betaceum*, a total number of 1,732,580 sequences were examined, of which 68,685 assembled contigs contained SSR motifs. The distribution of the different repeat type classes for both crops is detailed in Table 2.

### 3.2. SSR Variability

An amount of 100 SSR primers were tested in *S. quitoense* (see Supplementary Materials), 96 had validated PCR amplification and 41 were polymorphic in the screened Lasiocarpa species (Table 3). As detailed in Table 3, primer SSR transferability was total for 10 primers to the six Lasiocarpa screened species, and for 9 primers, it resulted in less than 50%. Among the wild species, the highest transferability rate was observed with the species *S. hirtum* and *S. candidum* (78.0% and 87.8%, respectively), while for *S. sessiliflorum*, the PCR rate was only 48.8%. Sequences of the 41 SSR primers are available in Supplementary Materials.

In the case of the cultivated, 14 primers showed polymorphism in *S. quitoense* cultivars. The primers detailed in Table 4 are, thus, useful SSR markers for *S. quitoense* genotyping. Among these, primers mSq012, mSq018, and mSq059 revealed three SSR alleles while the others only two.

Table 5 shows the diversity statistics of the 14 SSRs in *S. quitoense* and the Lasiocarpa species. Allele number was higher with primer mSq066 with 10 alleles while primer mSq041 only revealed four alleles. For four primers, the observed heterozygosity was higher in the cultivated *S. quitoense*, and the PIC values were in all cases lower than those obtained in wild species. The PIC mean value for the cultivated decreased from 0.64 to a value of 0.178.

Regarding *S. betaceum*, an amount of 110 primers were tested (Supplementary Materials). The amplification of the expected fragment in 75 primers was validated. Eight of these primers (mSb005, mSb082, mSb089, mSb09, mSb09, mSb102, mSb104, and mSb106) were potentially informative when the wild relative *S. unilobum* was screened. We observed a high homozygosity and no SSR variability among the screened varieties, even though a significant number of primers were tested.

### 3.3. Genetic Diversity Analysis

The diversity analysis of Lasiocarpas and S. quitoense varieties based on polymorphic SSR primers evidenced an average of 6.4 alleles per locus. The Msq066 primer was the most informative with a PIC value of 0.76, the average PIC of the analysis was 0.64, and the genetic diversity was 0.69 (see Table 5). In the diversity analysis of *S. quitoense* landraces, we observed up to four alleles per primer. The Msq012 primer was the most informative with a PIC value of 0.511, the average PIC was 0.178, and the genetic diversity was 0.219.

**Figure 1:**
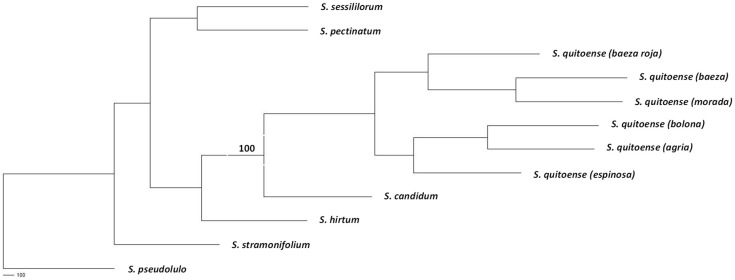
Unrooted majority rule consensus tree obtained with Phylip package and Consensus tree program showing bootstrap significant values with 1000 trees.

The consensus tree showed clades differentiated by species for each of the Lasiocarpas analyzed (Figure 1). The species *S. pseudolulo* appears to be the most divergent out of the remaining Lasiocarpa species. The remaining analyzed species are clearly distributed into three differentiated groups: *S. stramonifolium* forms a group separate from the rest; *S. sessiliflorum* and *S. pectinatum* form a second group; while *S. hitum*, *S. quitoense*, and *S. candidum* form a third group. The species *S candidum* is shown as being more closely related to *S. quitoense*. The *S. quitoense* cluster is clearly differentiated (supported by a 100 bootstrap value), distributing the cultivars into four subgroups: *Baeza roja* and *Espinosa* were distinguished from the *Baeza/Morada* and *Bolona/Agria* subgroups.

**Figure 2:**
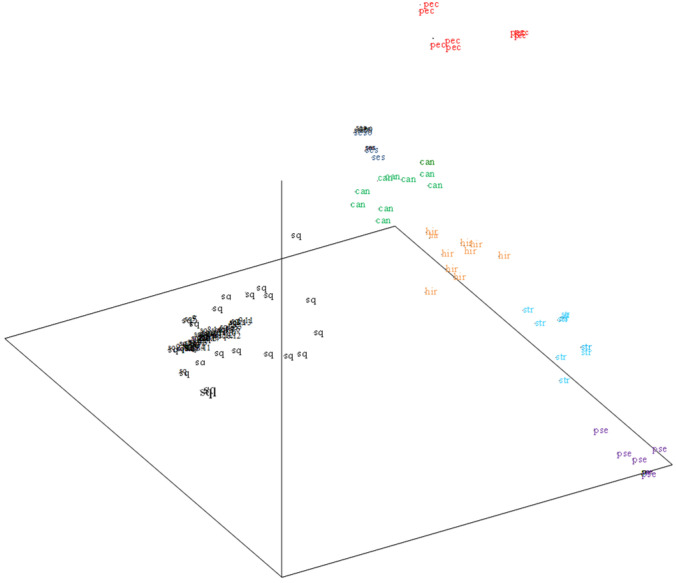
PCO analysis showing the dispersion of the 120 genotyped Lasiocarpa samples based on SSR polymorphism. *S. quitoense* genome. sq: *S. quitoense*; can: *S. candidum*; hir: *S. hirtum*; str: *S. stramonifolium*; pse: *S.pseudolulo*; pec: *S. pectinatum*.

The multivariate analysis (Figure 2) corroborates Lasiocarpa species differentiation from *S. quitoense* varieties. Bolona and Baeza roja appeared more differentiated from the rest of the *S. quitoense* samples. In terms of the Lasiocarpa species, *S. candidum*, *S. hirtum*, and *S. sessiliflorum* are located closer to *S. quitoense*. The analysis revealed that *S. stramonifolium*, *S.pseudolulo*, and *S. pectinatum* were more divergent.

In *S. betaceum* varieties, no polymorphic SSR primer was confirmed; only eight SSR primers were revealed to be polymorphic when the wild-related *S. unilobum* samples were included. The absence of SSR variability in *S. betaceum* is certainly due to the fact that the genetic base in this crop is very limited and SSR screening should include a high number of primers.

## 4. Discussion

The availability of high-throughput sequencing with NGS technology allowed the possibility to identify a substantial number of microsatellite sequences in *S. quitoense* and *S. betaceum* at lower cost and effort than precedent approaches [26,27]. The development of SSR technology in undervalued crops is an appropriate tool for Andean research programs in order to detect easily genetic variability or polymorphisms in local germplasm or segregant materials. In this first assessment, we screened a set of SSR primers for both species, generating a first set of useful primers for DNA genotyping.

In the exploratory genetic survey with the most representative *S. quitoense* varieties and the most used wild Lasiocarpa species in INIAP, 14 polymorphic SSR primers were detected, they showed high inter- and intraspecific variability with a PIC average value of 0.64 which is a higher value compared to a previously reported in *S. quitoense* (0.40) using heterologous SSR primers from *S. tuberosum* genome [6]. According to the interpretation of Botstein et al. [28], given the polymorphism index, markers with PIC values greater than 0.5 are considered highly informative, which is the case for the developed markers reported here (i.e., the PIC values of the 13 SSR primers are higher than 0.5). The 14 markers were used only to analyze *S. quitoense* varieties, and the PIC decreased to 0.178, which showed a reduced genetic base in the samples analyzed. This result reveals a reduced genetic base present in the cultivars compared to the wild species screened, probably the result of selective breeding derived from the domestication process. However, these results cannot be extrapolated to the entire *S. quitoense* crop existent [29], indicating that in order to infer data towards a population, the analyzed sample must be representative; thus, these results only reflect the usefulness of these SSR markers.

Regarding SSR transferability in other Lasiocarpa species, 41 useful primers were obtained. The transferred primers indicated that wild species have higher allelic diversity than *S. quitoense* varieties. This could be explained by the domestication process that many Andean fruit trees have suffered, most of which went from being wild to cultivated in a short space of time and therefore still share multiple alleles [30,31]. The diversity analysis clearly differentiated each Lasiocarpa species, as previously reported by Torres et al. [6] in a preliminary assessment with a reduced number of heterologous SSR primers. Among the SSR markers evaluated in *S. quitoense*, 10 showed heterozygous patterns, revealing 20 alleles among the cultivars. According to Lobo et al. [5], this result can be due to the low degree of domestication that some Andean fruit have suffered, where is possible to find heterozygosity as a result of survival mechanisms developed towards changing environmental conditions. However, a low genetic differentiation was observed between *S. quitoense* varieties, this finding agrees with the low rates of morphological, physiological, and genetic variability previously described [1,6,7,32]. Autogamy and selection by domestication could bring a decrease in allelic richness [6,8]. Fragmentation in natural populations and the replacement of varieties due to susceptibilities to diseases could explain the low genetic base in *S. quitoense* [8]. Other authors [5] have mentioned that due to there being a low degree of domestication in some Andean fruit trees, it is possible to find heterozygosity.

Diversity genetic analysis corroborated Lasiocarpa species differentiation from the cultivated *S. quitoense*. Torres et al. [6] argued that specific Lasiocarpa SSR markers will provide a better genetic relationship, which is the case in this study. This screening clearly evidenced that the Lasiocarpa species currently being used in genetic crosses with *S. quitoense* are genetically closer and are more related than other wild species.

In contrast, for *S. betaceum*, no polymorphic SSR primer was detected even when a significant number of SSR primers were screened. Only eight SSR primers were revealed to be polymorphic when including DNA samples from the wild *S.unilobum* species. Contrary to what was expected, the wild materials of *S. unilobum*, a species used as parents in the genetic breeding program in Ecuador, did not differ significantly from *S. betaceum*. This result agrees with a recent report [33], while a greater genetic distance between both species was previously reported [34]. In the same context, our SSR screen confirms the narrow genetic basis of the cultivars of *S. betaceum*. This result agrees with preliminary surveys which concluded that *S. betaceum* has a narrow and homogeneous genetic pool and homozygosity due to its autogamy system [15,26]. Domestication practices have caused the genetic erosion of the crop due to the limited use of varieties.

It is important to mention that the SSR bank sequences generated by NGS technology should be further explored for additional screening in this fruit crop. Applying other molecular tools based on NGS technology should be explored for additional screening in *S. betaceum*.

## 5. Conclusions

An important application of NGS technology is the development of molecular markers for understudied crops. SSR markers are one of the most informative and versatile markers used in plant genetic research. This study allowed us to efficiently identify a large number of SSR loci for *S. quitoense* and *S. betaceum* at a low cost. The set of SSR markers reported here will be used for surveying diversity in a larger collection of *S. quitoense* and Lasiocarpa germplasm. The SSR screening suggested a loss of alleles in the crop caused by domestication and the fragmentation of their populations. The validated SSR primers could be used with other molecular markers for developing a genetic map as a tool for breeding this important Andean fruit crop.

Meanwhile, the SSR sequence libraries generated for *S. betaceum* can be further explored for the screening of markers; however, other technologies such as Genotyping by Sequencing (GBS) should also be assessed in this crop.

## Figures and Tables

**Table 1:** Voucher information for biological material used in this research.

Species	Type	Variety or Group	INIAP ID or Source	Origin	Lab. Code
*S. quitoense*	Cultivated	Espinosa	INIAP ECU-3567	Pichincha-Ecuador	Sq1
*S. quitoense*	Cultivated	Naranjilla agria	INIAP ECU-3817	Bolívar-Ecuador	Sq2
*S. quitoense*	Cultivated	Naranjilla bolona	INIAP ECU-6235	Morona Santiago-Ecuador	Sq5
*S. quitoense*	Cultivated	Morada	INIAP Breeding program	Morona Santiago-Ecuador	Sq8
*S. quitoense*	Cultivated	Baeza	INIAP Breeding program	Morona Santiago-Ecuador	Sq9
*S. quitoense*	Cultivated	Baeza roja	INIAP Breeding program	Morona Santiago-Ecuador	Sq14
*S. candidum*	Wild	Lasiocarpa	INIAP ECU-13242	Not available	Sc
*S. hirtum*	Wild	Lasiocarpa	INIAP ECU-6242	Morona Santiago-Ecuador	Sh
*S. pectinatum*	Wild	Lasiocarpa	INIAP ECU-7875	Pastaza-Ecuador	Sp
*S. pseudolulo*	Wild	Lasiocarpa	INIAP Breeding program	Not available	Sps
*S. sessiliflorum*	Wild	Lasiocarpa	INIAP ECU-5552	Morona Santiago-Ecuador	Ss
*S. stramonifolium*	Wild	Lasiocarpa	INIAP Breeding program	Pichincha-Ecuador	St
*S. betaceum*	Cultivated	Puntón Anaranjado	INIAP Breeding program	Pichincha-Ecuador	PA
*S. betaceum*	Cultivated	Gigante Morado	INIAP Breeding program	Pichincha-Ecuador	GM
*S. betaceum*	Cultivated	Gigante Anaranjado	INIAP Breeding program	Pichincha-Ecuador	GA
*S. betaceum*	Cultivated	Puntón Morado	INIAP Breeding program	Pichincha-Ecuador	PM
*S. betaceum* × *S. unilobum*	Segregant	-	INIAP Breeding program	Pichincha-Ecuador	12 GR
*S. betaceum* × *S. unilobum*	Segregant	-	INIAP Breeding program	Pichincha-Ecuador	GT10P8
*S. betaceum* × *S. unilobum*	Segregant	-	INIAP Breeding program	Pichincha-Ecuador	GT13P25
*S. betaceum* × *S. unilobum*	Segregant	-	INIAP Breeding program	Pichincha-Ecuador	GT33P7
*S. betaceum* × *S. unilobum*	Segregant	-	INIAP Breeding program	Pichincha-Ecuador	Cruzam. 5
*S. unilobum*	Wild	progenitor	INIAP Breeding program	Pichincha-Ecuador	TUP1
*S. unilobum*	Wild	progenitor	INIAP Breeding program	Pichincha-Ecuador	25AP1
*S. unilobum*	Wild	progenitor	INIAP Breeding program	Pichincha-Ecuador	KUYP1
*S. unilobum*	Wild	progenitor	INIAP Breeding program	Pichincha-Ecuador	M15P1
*S. unilobum*	Wild	progenitor	INIAP Breeding program	Pichincha-Ecuador	SAN CARLOS

**Table 2:** Summary of sequencing and microsatellite (SSR) search in *S. quitoense* and *S. betaceum*.

SSR Search Results	*S. quitoense*	*S. betaceum*
Total number of sequences examined	1,400,090	1,732,580
Total size of examined sequences (bp)	274,369,691	296,038,400
Total number of identified SSRs	34,832	115,436
Number of SSRs containing sequences	31,759	68,685
Number of sequences containing more than 1 SSR	2370	30,063
Number of SSRs present in compound formation	2759	46,752
Distribution of different repeat-type classes		
Unit size	Number of SSRs	
2	18,349	16,385
3	13,385	91,665
4	1841	4348
5	856	274
6	401	2.764

**Table 3:** *S. quitoense* SSR primer transferability to six analyzed species of Lasiocarpa. The observed alleles for each species are indicated.

Primer	*S. sessiliflorim*	*S. stramonifolium*	*S. hirtum*	*S. candidum*	*S. pectinatum*	*S. pseudolulo*	PCR Rate
mSq_03	-	-	-	236, 239	-	-	16.7%
mSq_04	125	125, 170	125, 170	125, 161	125, 146	125	100.0%
mSq_06	158	182	140, 158	170	-	-	66.7%
mSq_08	-	173	167, 176, 215	167	158	164	83.3%
mSq_12	165	165	164	161	164	164	100.0%
mSq_13	145, 247	145, 247	145, 247	145	142, 145	142, 154	100.0%
mSq_16	247	-	235, 238, 256	247	244	238	83.3%
mSq_18	128, 137, 140, 143	137, 140, 143	137, 140, 143	122,140, 143	140, 143	-	83.3%
mSq_19	-	-	-	224, 227	206	200, 209	66.7%
mSq_20	195	-	195	201, 210	-	-	50.0%
mSq_21	-	-	136, 154	133	136	163	66.7%
mSq_23	-	-	-	107, 134	140	-	33.3%
mSq_24	104, 158	104, 149	125, 152	131	152	104, 143, 149	100.0%
mSq_26	105	111	105, 108	108	108	-	83.3%
mSq_27	104	104	98, 104	95, 104	89, 95, 104	98, 104	100.0%
mSq_28	153	150	153, 168	153, 162	153, 174	-	83.3%
mSq_29	-	-	145	-	-	-	16.7%
mSq_31	-	-	-	190	190	-	33.3%
mSq_33	-	-	-	143	134	122	50.0%
mSq_35	-	239	230, 242, 251	-	248	-	50.0%
mSq_36	110, 112	121	118	118, 130	112	115	100.0%
mSq_37	-	237	237, 249	234	246	-	66.7%
mSq_38	113, 146	113, 128	113, 128	113, 140	113, 128	113, 128	100.0%
mSq_40	209	206, 221	206, 221, 230	206, 218	206	206	100.0%
mSq_43	-	133	-	133	-	133	50.0%
mSq_44	118	114	126, 134	118	134	130	100.0%
mSq_46	-	-	231	-	-	231	33.3%
mSq_49	-	89	89	89, 91	89, 91	89	83.3%
mSq_50	160	182	150	150, 172	150	-	83.3%
mSq_51	-	-	235	247	-	-	33.3%
mSq_56	-	112	112	130	-	112	66.7%
mSq_57	-	158	168, 172, 176, 190	162	172	-	66.7%
mSq_58	-	140	144	144	176	160	83.3%
mSq_59	-	183	189, 191,193	179	179	179	83.3%
mSq_63	-	147	135, 137, 143	-	-	-	33.3%
mSq_66	142	156	150, 172	148	154	178	100.0%
mSq_68	176	-	-	172, 188	-	-	33.3%
mSq_84	82	82	-	-	88	90	66.7%
mSq_87	142	142	-	130, 142	150, 180	142, 162	83.3%
mSq_91	-	-	121	121, 135, 147	-	-	33.3%
mSq_93	-	129, 159	117, 141	133, 143	-	119, 137	66.7%
%	48.8%	68.3%	78.0%	87.8%	70.7%	58.5%	

**Table 4:** SSR markers useful for genotyping *S. quitoense* varieties.

Primer	Forward Primer	Reverse Primer	Motif	Size (pb)	Alleles
mSq006	TTACAGGGGAAGAGGGG	CGTATTTGTGTCTTATGTGGG	(AAT)11	170	170, 191
mSq012	TTCAAGTGTCAAGATTCAAG	AATTGTGTCAACTCTTACCC	(TTA)16	194	164, 194, 203
mSq016	CCATTATGCCTATCAATTCC	CTCGTCCCAAGAACAAAA	(AAT)12	256	244, 256
mSq018	TCTCCAAGATCCATGATT	AGGATGCTTCTTTTGATG	(ATT)13	143	140, 143, 247
mSq036	ACCAGCTTCAGAACATCAAA	GATTATTCTAGTAGCCGTCCCT	(AAT)9	118	118, 124
mSq040	AGTAAGTCACTCCAGTCTATTCA	CTAGTCCCCAAGCGAA	(ATT)11	221	209, 221
mSq049	ACAGGTATTACAAAGTCCACA	TTGGGAGCTTGTTTGTT	(AT)14	107	89, 107
mSq050	AATGCGAGGTGTGATAAATG	CATGTTGATGGTTTGGGA	(AT)15	172	172, 174
mSq058	AGATAGTCCTTCCCACCT	AAGAAAGTGATTTCGCC	(AT)14	162	156, 162
mSq059	TGAAGTCATAGCCACCAAC	CCACAAAGTTCCCTAATAAATC	(TA)15	195	179, 191, 195
mSq063	GCTTGAACAAACCAATTTCA	TTGCCACCAACTGAGGA	(TA)14	157	155, 157
mSq066	AGTCCCCTTGTATCTGGTG	GGAGAAAGGCAAGTGAGAG	(AT)15	162	162, 168
mSq068	TAAAATTAACACGACCCACA	AAGTGGCAAAGACGCA	(TA)17	188	186, 188
mSq091	CCGATTATGCAAGAAAGGT	GAGCTAGTTTAGCCTATTTTGGT	(AT)16	147	147, 165

**Table 5:** Genetic parameters calculated in 120 individuals of *S. quitoense* and Lasiocarpa accessions. Values for *S. quitoense* are differentiated in italics.

Primer	Genotypes	Availability Data (Na)	Alleles	Gene Diversity (He)		Heterozygosity (Ho)		PIC	
mSq006	6	113	6	0.65	*0.019*	0.06	*0.019*	0.61	*0.019*
mSq012	7	107	6	0.68	*0.592*	0.41	*0.936*	0.64	*0.511*
mSq016	10	112	6	0.73	*0.142*	0.13	*0.115*	0.69	*0.132*
mSq018	13	116	6	0.71	*0.492*	0.66	*0.875*	0.66	*0.371*
mSq036	9	114	8	0.63	*0.018*	0.13	*0.019*	0.61	*0.018*
mSq040	10	113	7	0.75	*0.497*	0.62	*0.925*	0.71	*0.374*
mSq049	4	115	4	0.55	*0.499*	0.46	*0.964*	0.49	*0.375*
mSq050	9	113	7	0.67	*0.073*	0.05	*0.000*	0.64	*0.070*
mSq058	7	106	7	0.75	*0.043*	0.00	*0.000*	0.72	*0.042*
mSq059	10	109	7	0.75	*0.203*	0.08	*0.061*	0.71	*0.189*
mSq063	6	110	7	0.65	*0.077*	0.05	*0.000*	0.58	*0.074*
mSq066	10	108	10	0.78	*0.117*	0.11	*0.042*	0.76	*0.110*
mSq068	6	108	5	0.71	*0.499*	0.01	*0.000*	0.66	*0.375*
mSq091	6	113	5	0.61	*0.019*	0.04	*0.019*	0.53	*0.019*
Mean	8	111.2	6.4	0.69	*0.23*	0.19	*0.28*	0.64	*0.178*

## Data Availability

SSR genotyping results can be found in the following drive: https://docs.google.com/spreadsheets/d/1mJM7VS2DkazMoC6hvb7kCtf8qeL59n8D/edit?usp=sharing&ouid=115434843326122486251&rtpof=true&sd=true (accessed on 27 February 2025).

## Supplementary Materials

The following supporting information can be downloaded at: https://www.mdpi.com/article/10.3390/plants14060874/s1, Figure S1. (A) *S. quitoense* plant and fruits. (B) *S. betaceum* plant and fruits; Table S1. SSR sequences Squitoense.

## References

[1] Heiser C.B. (1972). The relationships of the naranjilla, *Solanum quitoense*. Biotropica.

[2] Silva W., Gómez P., Sotomayor A., Viteri D., Ron L. (2016). Selección de líneas promisorias de naranjilla para mejorar la calidad de la fruta. Ecuad. Calidad..

[3] Carrera K., Isla H., Lidcay F., Cupull Santana R. (2018). Identificación de aislados de *Fusarium* spp. asociados a *Solanum quitoense* Lam en Pastaza, Ecuador. Centro Agrícola..

[4] Salazar-González C., Betancourth-García C. (2017). Reaction of genotypes of lulo (*Solanum quitoense* Lam.) to *Meloidogyne* spp. under field conditions. Cienc. Tecnol. Agropecu..

[5] Lobo M., Medina C.I., Delgado Paz O.A., Bermeo Giraldo A. (2007). Variabilidad morfológica de la colección colombiana de lulo (*Solanum quitoense* Lam.) y especies relacionadas de la sección Lasiocarpa. Rev. Fac. Nac. Agron. Medellin.

[6] Torres A.F., Arias A.S., Arahana V., Torres M.L. (2008). Preliminary Assessment of Genetic Diversity and Phenetic Relations for Section by Means of Heterologous SSR Markers. Crop Sci..

[7] Soria J. (1997). Genetic improvement of the “naranjilla” (*Solanum quitoense* Lam.) through interspecific crosses. Utilization and Management of Phyto-Resources.

[8] Fory Sánchez P.A., Sánchez Mosquera I., Bohórquez Cháux A., Ramírez H., Medina Cano C.I., Lobo Arias M. (2010). Genetic variability of the Colombian collection of lulo (*Solanum quitoense* Lam.) and related species of section Lasiocarpa. Rev. Fac. Nac. Agron. Medellín.

[9] Díaz Granda L., Canto Sáenz M., Alegre Orihuela J., Camarena Mayta F., Julca Otiniano A. (2017). Sostenibilidad social de los subsistemas productivos de tomate de árbol (*Solanum betaceum* Cav) en el Cantón Guachapala, Provincia de Azuay-Ecuador. Ecol. Apl..

[10] Villares M., Sánchez J., Viera W., Soria N., Sotomayor A., Yanez D., Martínez E. (2018). Caracterización morfológica de frutos de tomate de árbol (*Solanum betaceum* Cav.) de una población segregante. Rev. Investig. Talent..

[11] Moreno-Miranda C., Molina J.I., Ortiz J., Peñafiel C., Moreno R. (2020). Cadena de valor en la red de tomate de árbol (*Solanum betaceum*) en Ecuador. Agron. Mesoam..

[12] Criollo H., Insuasti K., Delgado W. (2016). Regeneración in vitro de plántulas de tomate de árbol (*Solanum betaceum* Cav. Sendt.). Rev. Colomb. Cienc. Hortícolas.

[13] Caruso G. (2015). Diversidad genética. Importancia y aplicaciones en el mejoramiento vegetal. Lhawet.

[14] Sari D., Sari H., Ikten C., Toker C. (2023). Genome-wide discovery of di-nucleotide SSR markers based on whole genome re-sequencing data of *Cicer arietinum* L. and *Cicer reticulatum* Ladiz. Sci. Rep..

[15] Peñafiel N.L., Arahana V.S., Torres M. (2009). Evaluación de la variabilidad genética del tomate de árbol (*Solanum betaceum* Cav) en los cultivos de tres provincias del Ecuador por medio de marcadores microsatélites. ACI Av. Cienc. Ing..

[16] Rodríguez E.F., Martínez R., Lobo M., Barrero L.E. (2010). Genetic variation in the Solanaceae fruit bearing species lulo and tree tomato revealed by Conserved Ortholog (COSII) markers. Genet. Mol. Biol..

[17] Acosta-Quezada P.G., Raigón M.D., Riofrío-Cuenca T., García-Martínez M.D., Plazas M., Burneo J.I., Prohens J. (2015). Diversity for chemical composition in a collection of different varietal types of tree tomato (*Solanum betaceum* Cav.), an Andean exotic fruit. Food Chem..

[18] Souza H., Muller L., Brandão R., Lovato M. (2012). Isolation of high quality and polysaccharide-free DNA from leaves of Dimor-384 phandra mollis (Leguminosae), a tree from the Brazilian Cerrado. Genet. Mol. Res..

[19] Simpson J.T., Wong K., Jackman S.D., Schein J.E., Jones S.J., Birol I. (2009). ABySS: A parallel assembler for short read sequence data. Genome Res..

[20] Thiel T. (2001). MISA-MIcroSAtellite Identification Tool, Version 1.0. MISA-MIcroSAtellite Identification Tool.

[21] Kang H., Cho Y., Yoon U., Eun M. (1988). Rapid Genotypes Analysis Using DNA Extracted from Single Half Seed of Rice Plant. Mol. Biol. Reporter..

[22] Morillo E., Miño G. (2011). Marcadores Moleculares en Biotecnología Agrícola: Manual de Procedimientos y Técnicas en INIAP.

[23] Liu K., Muse S.V. (2005). PowerMarker: An integrated analysis environment for genetic marker analysis. Bioinformatics.

[24] Rohlf F.J. (1992). NTSYS-pc. Numerical Taxonomy and Multivariate Analysis System.

[25] Felsenstein J. (1993). PHYLIP (Phylogeny Inference Package).

[26] Taheri S., Lee Abdullah T., Yusop M.R., Hanafi M.M., Sahebi M., Azizi P., Shamshiri R.R. (2018). Mining and development of novel SSR markers using next generation sequencing (NGS) data in plants. Molecules.

[27] Zhu J., Zhang J., Jiang M., Wang W., Jiang J., Li Y., Zhou X. (2021). Development of genome-wide SSR markers in rapeseed by Next Generation Sequencing. Gene.

[28] Botstein D., White R.L., Skolnick M., Davis R.W. (1980). Construction of a genetic linkage map in man using restriction fragment length polymorphisms. Am. J. Hum. Genet..

[29] Otzen T., Manterola C. (2017). Técnicas de Muestreo sobre una Población a Estudio. Int. J. Morphol..

[30] Gepts P. (2002). A comparison between crop domestication, classical plant breeding, and genetic engineering. Crop Sci..

[31] Bernal N., Ocampo J., Hernández J. (2014). Caracterización y análisis de la variabilidad Genética de la granadilla (*Passiflora ligularis* juss.) en Colombia empleando marcadores microsatélites. Rev. Bras. Frutic..

[32] Sosa P.A., Batista Hernández F., Bouza Carrelo N., González Pérez M.Á. (2002). La conservación Genética de las Especies Vegetales Amenazadas.

[33] Zambrano-Fierro P. (2020). Caracterización Molecular de un Grupo de Híbridos Provenientes del Cruzamiento (Solanum betaceum Cav. x Solanum unilobum) del CADET. Tumbaco.

[34] Leiva L.E. (2008). Caracterización de dos Poblaciones Segregantes de Tomate de Árbol (Solanum betaceum) Mediante Marcadores Moleculares AFLP. Bachelor’s Thesis.

