# Supplementary material for: Genetic Assessment in the Andean Tropical Fruits Solanum quitoense Lam. and S. betaceum Cav.: Efforts Towards a Molecular Breeding Approach
Source: Plants (Basel). 2025 Mar 11;14(6):874. doi: 10.3390/plants14060874 (PMC11944818; doi:10.3390/plants14060874)

PLANTS

Genetic assessment in the Andean tropical fruits ***Solanum quitoense*** and ***S. betaceum***: efforts towards a molecular breeding approach

Eduardo Morillo[1], Johana Buitron[1], Denisse Yanez[1,2], Pierre Mournet[3] , Wilson Vásquez-Castillo[2] and Pablo Viteri[1]

[1]Estación Experimental Santa Catalina-INIAP Quito, Ecuador [2] Universidad de las Américas (UDLA), Ingeniería Agroindustrial y Alimentos, Quito, Ecuador [3]CIRAD-BIOS, UMR 1334 AGAP, Montpellier, France

Figure S1A. *S. quitoense* plant and fruits

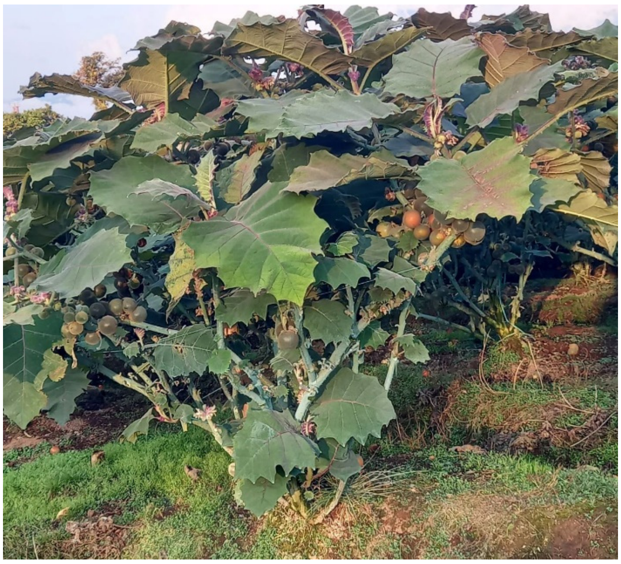

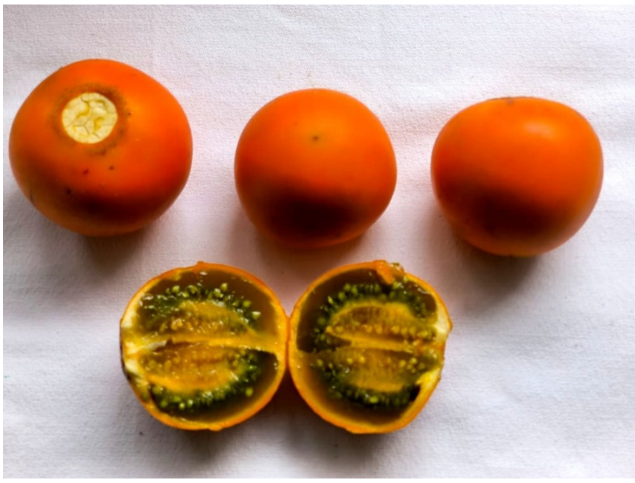

Figure S1B: *S. betaceum* plant and fruits

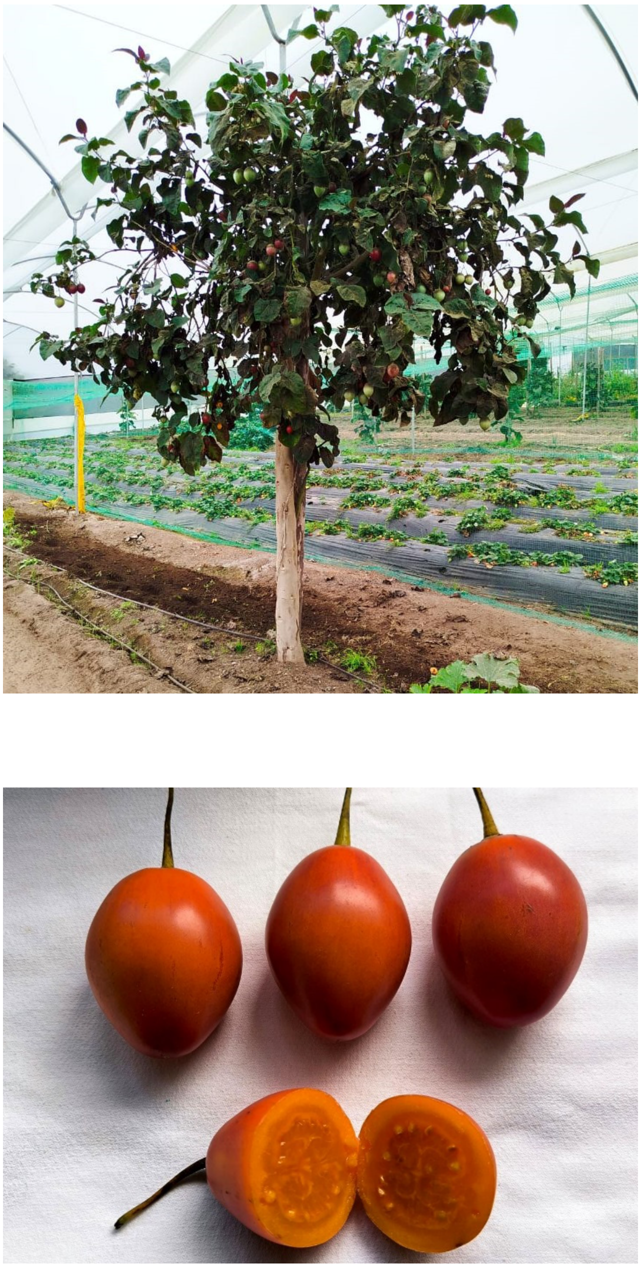

Supplement: Supplementary file 1 [file plants-14-00874-s001.zip › plants-3431391-Supplementary_figures.pdf]
